# Divergent stakeholder perspectives on antimicrobial resistance and One Health in Ghana: a qualitative study

**DOI:** 10.1186/s44263-026-00319-1

**Published:** 2026-08-19

**Authors:** Maresa Neuerer, Carine Baxerres, Anastasia A. Seferiadis, Daniel K. Arhinful, Alexander Amponsah, Leslie Mawuli Aglanu, Valérie R. Louis, John H. Amuasi, Aurélia Souares

**Affiliations:** 1 Heidelberg Institute of Global Health, University of Heidelberg, Im Neuenheimer Feld 130.3, Heidelberg, 69120 Germany; 2 Laboratoire Population Environnement Développement, Aix Marseille Université, Institut de Recherche pour le Développement, 3 Place Victor Hugo, Marseille, 13003 France; 3 Noguchi Memorial Institute for Medical Research, University of Ghana, P.O. Box LG 581, Legon, Accra Ghana; 4 Bernhard Nocht Institute for Tropical Medicine, Bernhard-Nocht-Str. 74, Hamburg, 20359 Germany; 5 Kumasi Centre for Collaborative Research in Tropical Medicine, PMB UPO, Kumasi, Ghana; 6 University Medical Centre Groningen (UMCG), University of Groningen, Groningen, 9713 GZ The Netherlands; 7 School of Public Health, Kwame Nkrumah University of Science and Technology, PMB UPO, Kumasi, Ghana; 8 German Centre for Infection Research (DZIF), Heidelberg Site, Heidelberg, Germany

**Keywords:** Antimicrobial resistance, Antibiotics, One Health, Qualitative study, Perception, Knowledge, Institutional actors, Community members, Farmers

## Abstract

**Background:**

Antimicrobial resistance (AMR) is a global threat to human, animal, and environmental health driven by natural processes and human practices. One Health, originating from biomedical concerns about zoonoses, links human, animal, and ecosystem health and frames AMR as a systemic issue.

**Methods:**

This qualitative study, conducted in the Asante Akim North Municipality of Ghana, aimed to assess how community members, farmers, and institutional actors across animal, environmental, and human health sectors understand AMR and One Health in the context of AMR. Data were collected between November 2021 and October 2023 through ethnographic fieldwork (participant observation, unstructured and in-depth interviews) and socio-epidemiological methods (key-informant interviews, customer-exit interviews). Data were analysed using thematic analysis in NVivo 14, with integration through collaborative cross-coding and interdisciplinary team review.

**Results:**

A total of 103 participants were included. Three overarching themes emerged: divergent awareness of AMR, contrasting explanatory models of antibiotic ineffectiveness, and uneven recognition of cross-sectoral health interconnections. Community members and farmers had little known prior exposure to AMR, and thus rarely perceived it as a problem. After the concept was explained, they drew on experiential models that attribute antibiotic ineffectiveness to physiological differences such as blood-drug incompatibility or the body “getting used” to drugs. Institutional actors framed AMR as an urgent global health threat with health and economic consequences, using language closely aligned with international policy discourse, yet reported limited personal experience of it. Regarding cross-sectoral dimensions of AMR, community members and farmers described links between health, animals, and environments through everyday concerns about hygiene, close contact with animals, and disease transmission. These insights resonate with One Health principles without using the term itself. Institutional actors strongly endorsed One Health at the policy level but described fragmented implementation and the persistent marginalisation of the environmental health sector.

**Conclusions:**

These contrasting explanatory models carry implications for AMR intervention design. Integrating pluralistic explanatory models may strengthen the contextual relevance and sustainability of One Health-based AMR interventions.

**Supplementary Information:**

The online version contains supplementary material available at 10.1186/s44263-026-00319-1.

## Background

Antimicrobial resistance (AMR) is a global challenge to animal, environmental, and human health [1]. While it occurs naturally through biological, molecular, or environmental vectors, it is also amplified by human activity across multiple sectors [2]. Contributing factors include among others antibiotic overuse in people and animals, discharge of antimicrobial residues into the environment, and use of broad-spectrum antibiotics when infection causes are unclear [1, 3]. Additionally, weak policy enforcement and limited public awareness bolster the emergence of AMR [4, 5]. Addressing AMR therefore requires coordinated action across animal, environmental, and human health systems, as resistance-driving bacteria do not respect sectoral boundaries. One Health is promoted by international organisations and institutions, and describes the interdependence of the health of animals, ecosystems, and humans [6]. While it originated from biomedical concerns focused on human health in relation to zoonoses [7, 8], it has also been taken up in more expansive registers, including philosophical perspectives that emphasise its relational and ethical dimensions [9].

AMR concerns all countries, but literature indicates that effects tend to be more significant in low- and middle-income countries (LMICs) due to already existing inequities such as fragile healthcare systems and insalubrious environmental conditions [10, 11]. Globally, western sub-Saharan Africa recorded the highest mortality rate attributed to and associated with AMR in 2021 [12]. However, more deaths are still attributed to limited or delayed access to existing high-quality routine antibiotics in LMICs than to AMR itself [13, 14]. In 2018, Ghana adopted a One Health-based National Action Plan and established the AMR Policy Platform and Secretariat [15, 16].

As political and institutional commitment to addressing AMR continues to grow with an increased promotion of the One Health approach, it has become important to understand how different stakeholders conceptualise AMR. Across a range of health issues, such as malaria, maternal health, or diabetes, to healthcare management strategies, including universal health coverage and performance-based financing, the literature has extensively documented discrepancies between the perceptions and practices of policymakers, healthcare providers, and patients, revealing that policy adoption does not translate directly into shared understanding or coordinated action [17, 18]. In the case of AMR, despite growing efforts at the policy level, there remains limited understanding of how community members, farmers, and institutional actors across the animal, environmental, and human health sectors conceptualise the issue on the ground [19]. Bridging this knowledge gap is essential for developing contextually relevant interventions that are grounded in community participation. Despite the adoption of national action plans in many countries, including Ghana, implementation has been limited [16], in part because top-down approaches have not adequately accounted for the perspectives of different stakeholder groups.

To inform the participatory co-design of an AMR intervention in Ghana, this study examined how community members, farmers, and institutional actors understand AMR and whether they recognise the interconnection between animal, environmental, and human health as relevant to antibiotic use and resistance.

## Methods

### Study design

This study is part of the multidisciplinary AMR-B-CHANGE project, that aims to develop an AMR intervention through a participatory approach [20]. We employed a combination of anthropology and socio-epidemiology methods to generate baseline data on understandings of AMR and One Health in the context of AMR.

An ethnographic study included immersion in communities and semi-intensive farms (500-8,000 birds), participant observations, unstructured and in-depth interviews (IDIs) between November 2021 and November 2022. This data was complemented with a socio-epidemiology component that included key informant interviews (KIIs) with institutional actors between August 2022 and April 2023, IDIs with community members and farmers in October 2023. Combining these two methodological traditions allowed for triangulation of theories, methods, data, analyses, and investigators. This study adhered to the COREQ reporting guidelines; the completed checklist can be found in Supplementary Material 1: COREQ Checklist.

### Study setting

The study was conducted in Ghana in the semi-urban and rural Asante Akim North Municipal Assembly, with Agogo as the administrative capital. The district covers 1,160 km² and has 68,186 inhabitants, 53.5% of whom live in rural areas, predominantly youth and middle-aged adults [21]. Agriculture employs 72.7% of the workforce, mainly in smallholder cultivation of plantain, yam, rice, oil palm, and vegetables, while livestock production, primarily poultry, constitutes 21.7% of agricultural activities [21].

This study site was selected because it serves as the primary field research setting of the Kumasi Centre for Collaborative Research in Tropical Medicine (KCCR), one of the Ghanaian partner institutions in this study. KCCR routinely conducts clinical and epidemiological research in the area, including hospital-based AMR surveillance, which provided a foundation for the present study’s community- and farm-level enquiry. Selecting a site with existing research infrastructure and established community relationships facilitated access to participants across sectors and enabled the integration of the study within ongoing AMR data collection efforts.

### Study participants

Within the anthropology component, we conducted an ethnographic study, involving participant observations, IDIs, and informal interviews, among communities in two areas: the semi-rural town of Agogo and the remote village of Bebome, one of the five rural zones of Agogo municipality. We also studied semi-intensive poultry farms (500-8,000 birds; established mid-2000’s to date) in Agogo, where this activity was concentrated. We aimed to compare access to antibiotics, health seeking behaviour, and related perceptions between populations living in a town with relatively good access to healthcare services, basic necessities, and transportation, and those living in a small, difficult-to-reach village. Thirty households, 15 in Agogo and 15 in Bebome, were selected based on income-generating activities, material assets (house, vehicle, appliances, communication devices), and presence of at least one child under 10 years, as we wanted to assess antibiotic use among adults and children, with the aim of reaching diversity across these criteria. Within these households, we conducted semi-structured IDIs with 30 mothers, 13 fathers, and 2 grandmothers. Additionally, we carried out ethnographies on six semi-intensive poultry farms, with 14 IDIs conducted among farm owners and employees. The anthropology fieldwork was conducted by CB, AAS, and AA.

Within the socio-epidemiology component, we held semi-structured KIIs with 25 institutional actors from the animal, environmental, and human health sectors, defined as individuals in organisations involved in AMR-related management, regulation, or research (e.g. ministries, agencies, academic institutions, international organisations, research bodies). Respondents were selected through purposive and snowball sampling to cover all One Health sectors at district, regional, and national levels. Additionally, we used a customer-exit approach [22] to recruit 11 community members and 8 farmers who had just bought antibiotics. Detailed participant demographics, including final analytical sample size by discipline and interview type, are presented in Supplementary Material 2: Table S1.

### Data collection procedures

We obtained institutional authorisation from relevant authorities and community authorisation through meetings with traditional leader representatives and local authorities (assembly members). All respondents were informed about the project and gave informed consent before their participation. Regular debriefings were held during data collection. Observation and interview guides, and information sheets for both study components are available in Supplementary Material 3: Guides and information sheets.

Within the anthropology component, a Ghanaian research assistant (AA) lived in Agogo for one year. He rented a room in a shared house and used daily activities (shopping, laundry, cooking, administrative and healthcare tasks) to conduct observations and informal interviews with residents in both study areas, guided by the research questions. His wife lived with him and gave birth to their first child, further facilitating his immersion in local realities. AA wrote detailed daily field diaries after observations and informal interviews. Following this, he conducted IDIs within households, one per person, lasting 45–90 min each. He applied a similar approach in Bebome, where he stayed for one month in quarters reserved for visitors. The choice of 30 families, 15 in Agogo and 15 in Bebome, was a reasoned choice, grounded in the prior year of participant observation and informal interviews. This allowed us to include transcripts of IDIs alongside field diaries, consisting of observations and informal interviews, and achieve data saturation.

Additionally, CB, AAS, and AA conducted ethnographies on six semi-intensive poultry farms for around 2 months in each farm. They spent 5–7 h per day working on the farm alongside workers, feeding birds, collecting eggs, cleaning coops, and accompanying the owner or employees to purchase feed and, occasionally, poultry medicine. At the end of each ethnography, CB, AAS, and AA conducted IDIs, lasting 43–222 min, with farm owners and employees. Fieldwork was carried out by AA mostly in Twi, and by CB and AAS in English.

For the socio-epidemiology component, we conducted 20 KIIs with institutional actors face-to-face and four online via video or audio calls depending on the network quality, using an institutional Zoom account (Zoom Video Communications, Inc., San Jose, CA, USA). Three KIIs were not recorded because of background noise; notes were taken instead. Two of the face-to-face KIIs were conducted with two and three respondents due to participant preferences. KIIs were conducted in English by AAS and MN for 20–98 min. For the exit interviews with community members and farmers, MN and AA were stationed at two Over-the-Counter Medicine Seller’s outlets, two pharmacies, one community health centre, two veterinary shops, and one informal veterinarian at a farmer’s market. Sales staff were asked to notify them when a customer purchased antibiotics, after which the customer was approached and invited to participate. This recruitment strategy reflects an analytical distinction between farmers and community members: farmers were distinguished by their occupational relationship with livestock kept for income and their associated exposure to veterinary antibiotic use, which was expected to produce distinct AMR-related practices and perceptions. IDIs were conducted with community members and farmers in Twi, lasting 20–60 min. One farmer and one community member discontinued their participation due to discomfort and limited proficiency in Twi. All interviews were audio-recorded using the built-in voice recorders of the personal smartphones of the researchers who collected data, all placed in flight mode to protect confidentiality.

Across both the anthropological and socio-epidemiological components, interviews followed the same progression for exploring understanding of antibiotics and AMR. Interviewers did not begin with the biomedical category ‘antibiotics’; instead, they first responded to participants’ spontaneous mentions of specific medicines by their locally recognised Twi names (e.g., *tupaye* red and yellow for oxytetracycline, *hyee kuro* for penicillin V), as well as any English (e.g., amoxicillin) or abbreviated international non-proprietary names (e.g., amoxi, cipro) participants used. They then discussed the health problems these medicines were taken for (e.g., skin wounds, internal ailments, stomach aches, coughs, fevers). Only later was the English term ‘antibiotics’ introduced to understand whether and how participants grouped (some of) these products into a single category. To explore AMR, interviewers avoided the acronym entirely and initially alluded to the phenomenon without naming it, using questions such as “do medications sometimes fail to work or work less effectively?” or “do health problems sometimes return after being treated?”. The term ‘antimicrobial resistance’ was then introduced and explained using a standardised, accessible formulation developed by the anthropology team, conveying that an antibiotic may lose its effectiveness regardless of dosage, without reliance on biomedical jargon. This explanation then served as a basis to further explore respondents’ own understandings of the issue. Throughout AMR discussions questions reflecting One Health principles, the interconnectedness of animal, environmental and human health, were woven in, though the term ‘One Health’ itself was never used. One Health was examined specifically in the context of AMR, exploring whether participants recognised the interconnection between human, animal, and environmental health as relevant to antibiotic use and resistance, rather than as a standalone evaluation of One Health understanding.

### Data management and analysis

All interviews were transcribed verbatim and, when conducted in Twi, translated into English during transcription. Data were analysed in two distinct NVivo 14 projects (version 14.23.4, Lumivero, Denver, CO, USA) using thematic analysis [23]. Participants from both disciplinary components were categorised into three analytical groups: community members, farmers, and institutional actors (identified by sector).

In the ethnographic study, interviews and field diaries, including observations and unstructured interviews, were coded through a mixed approach (deductive and inductive) by CB, AAS, and AA. A thematic analysis guide was developed collectively during a one-week workshop, and the team met weekly to review and refine emerging codes and interpretations. In the socio-epidemiological study, initial codes were developed both deductively, informed by existing AMR literature, and inductively, based on emerging patterns in the data. A sample of three interviews, one per stakeholder group (animal health, environmental health, human health), was double coded by MN and a trained research assistant, and discrepancies were resolved through structured discussion until consensus was reached. All transcripts were then coded by MN simultaneously using this unified framework, without prior separation by participant group, meaning that synthesis across sources and participant categories was embedded throughout the coding process. The coding framework and emerging themes were further validated through iterative review with the multidisciplinary and international research team.

Following independent analysis of each corpus (anthropology and socio-epidemiology), integration was conducted at the coding level within NVivo 14. The anthropology team shared all data segments coded under “perception of AMR” with the socio-epidemiology team. This was one of the 116 sub-themes of the anthropology team’s NVivo project and encompassed coded material with 256,230 characters (including spaces). These segments were imported into the existing socio-epidemiology coding framework, where they were re-coded into its more granular structure to ensure consistency across the combined dataset. Where ethnographic material introduced dimensions not accommodated by the existing codes, new sub-codes were created. The interdisciplinary team held recurring analytical meetings to examine convergences and divergences across data sources. Throughout the results, data from field diary entries, including observations and unstructured interviews, are indicated as such, and all quotes, whether derived from anthropological or socio-epidemiological data, are attributed by participant type (community member, farmer, institutional actor). Institutional actors are further identified by sector (human health, animal health, or environmental health).

### Researcher reflexivity

MN is a female, doctoral candidate working within the AMR-B-CHANGE project. Her approach to AMR is informed by a holistic understanding of health, social determinants of health, and underlying structures of health behaviour. CB is a female, senior researcher, with extensive experience in interdisciplinary research projects. This research was the first that she supervised on AMR, and she had no specific preconceptions about the issue of AMR before conducting the study. AAS is a female, post-doctoral researcher. Her approach to AMR was devoid of any preconception, engaged in a knowledge co-construction approach attentive of sources of knowledge outside of academia. AA is male, Ghanaian, a native Twi speaker, and a master’s graduate. He was recruited for the purpose of this study as a research assistant. He had previously collected data on mental health but never on AMR or pharmaceuticals.

As researchers from Germany and France working in Ghana, MN, CB, and AAS acknowledge their outsider status, reinforced by their limited proficiency in Twi (restricted to greetings and simple interactions). All researchers involved in data collection (MN, CB, AAS, AA) lived in a rented room within compound houses in Agogo during their respective data collection periods and engaged in daily community activities, which supported sustained immersion and helped reduce social distance between the research team and community members.

## Results

### From unfamiliar concept to recognised problem

Across community members and farmers, the concept of ‘antimicrobial resistance’ was almost entirely unfamiliar, whereas institutional actors in animal, environmental, and human health described AMR as an important topic within their professional fields. Community members uniformly reported that they had never heard the term, and when AMR was explained, many questioned whether such a phenomenon could exist, explaining that *“these drugs [antibiotics] work for everyone […] and so I have not heard it before” (community member*,* female*,* 18–29 years*,* housewife)*. Among farmers, awareness was similarly limited: most had not encountered the term ‘antimicrobial resistance’, although one farmer recalled hearing about it from a veterinary officer and another had attended a workshop on antimicrobial resistance. *“I hear it [AMR]*,* especially from the vet officers*,* yes*,* because sometimes when they meet us*,* they talk about some of these things. […]. He has been telling me how most of these drugs no longer work […] because they have been abused” (farmer*,* male*,* 41–51 years*,* semi-intensive poultry farm)*.

In contrast, animal health professionals consistently portrayed AMR as a major problem affecting both veterinary practice and animal production systems, and environmental health actors framed AMR as a relatively new but increasingly important topic within their sector. One participant highlighted that, compared to animal and human health, the environmental sector had initially been *“on holiday enjoying” (institutional actor*,* female*,* > 52 years*,* environmental health*,* policy*,* national level)* when AMR discussions began. Human health respondents described AMR as *“a big problem because we do see high rates of resistance” (institutional actor*,* male*,* > 52 years*,* human health*,* research*,* regional level)*. Across institutional actors, several linked their awareness of AMR to prior exposure through university training, research projects, or participation in national and international conferences, networks, workshops or training programmes, which they explained as resulting in broader awareness of its consequences.

### A future threat versus a current, invisible crisis

Perceptions also diverged regarding when and how AMR was seen as a problem: following an explanation of the concept, community members and farmers tended to frame it as either non‑existent or a potential future issue, while institutional actors described AMR as an urgent, ongoing crisis. Among community respondents, most stated that *“it is not a problem” (community member*,* male*,* > 52 years*,* chemical shop owner)*. However, a few community members expressed concern about the potential future challenge of ineffective medication: *“the same way it is a problem for me*,* it could be a problem for someone else [referring to the future]. […] So*,* if someone takes it and doesn’t get healed*,* he will be worried” (community member*,* male*,* 30–40 years*,* crop farmer)*. Farmers similarly located AMR in the future; after AMR was explained to them, many acknowledged that resistance could exist but emphasised that, apart from one farm where a veterinarian had diagnosed AMR, they had not yet experienced it directly. A few farmers described instances in which antibiotics did not work for themselves or colleagues, but these episodes were not interpreted as evidence of AMR.

Institutional actors, by contrast, depicted AMR as both pervasive and difficult to see. Among animal health respondents, AMR was described as *“the silent pandemic*,* that is not getting the attention that a pandemic really requires” (institutional actor*,* male*,* 41–51 years*,* animal health*,* practitioner*,* regional level)*. They repeatedly highlighted low awareness among farmers and other policymakers, positioning limited recognition outside their own professional communities as a key barrier to action on AMR: *“a lot of people don’t know much about [it]” (institutional actor*,* male*,* > 52 years*,* animal health*,* research*,* regional level)*. Environmental health actors similarly described AMR as an important global issue gaining momentum, with many organisations and institutions pushing the work on AMR forward. While human health respondents stressed that AMR is a big problem, one respondent pointed out the challenge that AMR *“is not visible. […] For you to say*,* this patient really died of resistance is difficult because of all the comorbid[ities]” (institutional actor*,* male*,* 41–51 years*,* human health*,* policy*,* national level)*. Several respondents pointed out that while there is awareness among human health professionals, AMR is not a priority: *“AMR does not kill like HIV*,* […] which is sexy and makes everybody stand up. AMR is like a frog in boiling water. […] [If] you put a frog in cold water [and] put it on fire*,* it is warm water […] before you [the frog] realise you are cooked. [That’s what] AMR is – it’s slow*,* and nobody cares” (institutional actor*,* male*,* 41–51 years*,* human health*,* policy*,* national level)*. Very few institutional respondents reported personal or family health complications related to AMR. One who had such experience described how it reshaped his understanding of the problem, underscoring how the perceived invisibility of AMR is partly sustained by the rarity of directly attributable, lived experiences: *“If you have never come in contact with resistant bacteria*,* you will not understand this. […] I have come in contact with it – my mom*,* four years ago.” (institutional actor*,* male*,* 41–51 years*,* human health*,* policy national level)*.

### Mismatch between drug, blood, and disease

After the concept of ‘antimicrobial resistance’ was introduced, several community members described it as resulting from individual physiological differences, explaining that *“not all drugs work for people. Some people’s* blood *cannot contain some drugs” (community member*,* female*,* > 52 years*,* trader)*. “*When he gets a wound and he puts it [red and yellow*,* a local term for tetracycline capsules that are half-red and half-yellow] on the wound*,* it doesn’t work*,* then it starts oozing fluids. […] But when you put the penicillin V on the wound*,* it helps.” (community member*,* female*,* 18–29 years*,* crop farmer)*.

Several farmers articulated closely related ideas, linking resistance to blood type, expired medications, or the “strength” of particular diseases: *“Maybe the drug I bought wasn’t strong enough […] or the disease was stronger than the drug and it didn’t work like it was supposed to.” (farmer*,* male*,* > 52 years*,* small-scale mixed farm)*. These explanations, like community members’ accounts of “mixing of blood and medication”, locate resistance in a mismatch between the drug, the body, and the disease. Some farmers also described the need to change or alternate medicines over time to restore effectiveness, reinforcing an understanding of AMR as a problem of diminishing medicine “strength” in relation to the body and disease.

### Consequences of antibiotic use practices

Community members and farmers also understood AMR as linked to specific patterns of antibiotic use, with several community respondents explaining that high use of antibiotics causes the antibiotic to adapt to the body and become ineffective. *“When you overuse it [antibiotic]*,* the day you are really sick*,* […] it does not work for you” (community member*,* 30–40 years*,* trader)*. Some respondents related AMR to personal or family members’ experiences with ineffective malaria treatment, which they attributed to incompatibility between the medication and the individual’s physiology. Other respondents linked AMR to their experience with ineffective medication for diabetes, hypertension, and pain, explaining that *“when you take [a drug] a lot*,* it mixes with your blood and no longer work[s] […] to cure what you were taking it for” (community member*,* > 51 years*,* retired)*. In a similar way, some also referred to the reduced effectiveness of paracetamol. These examples conceptualise resistance as a broader phenomenon extending beyond antibiotics. Based on personal experience and explanations from doctors, one community member linked not completing treatment courses to *“parasites [that] are resistant to that [medicine]” (community member*,* > 52 years*,* crop farmer)*.

Several farmers similarly attributed resistance to disregarding dosages, prophylactic use, and overuse of antibiotics in animals. *“Most drugs no longer work. It doesn’t work at all because they have been abused” (farmer*,* male*,* 41–51 years*,* semi-intensive poultry farm)*. One farmer explained that with repeated use *“the system will get used to that thing […]. If we overuse it*,* it becomes ineffective” (farmer*,* male*,* 18–29 years*,* small-scale mixed farm)*. This notion of biological adaptation due to overuse was also reflected in field diary observations, where similar conceptualisations were noted during informal interactions at farming sites (field diary, Agogo, 9 Sept 2021). Some farmers suggested that alternating antibiotics was necessary to maintain treatment effectiveness, indicating both awareness of diminished efficacy and attempts to manage it through rotation. One farmer who had attended an AMR workshop described frequent antibiotic use as making bacteria stronger and reported that this understanding had led him to avoid prophylactic use.

Institutional actors in the animal and human health sectors provided more elaborated accounts that nonetheless foregrounded similar use-related drivers. Almost all animal health professionals described AMR as *“a multi-factored process [where] man […] has a role to play [in everything]*,* [from] regulation […] [to] attitude […]. Because that moment we would make a mistake*,* [it can] bring a lot of big issues” (institutional actor*,* male*,* 30–40 years*,* animal health*,* policy*,* national level)*. Misuse of antibiotics by farmers played a central role in this process: *“I think one of the things that are related to that is the indiscriminate use of antibiotics by our farmers and our animal handlers. Because when they see anything*,* they just give*,* they just give” (institutional actor*,* male*,* 30–40 years*,* animal health*,* practitioner*,* regional level)*.

Environmental health professionals, although less directly engaged in AMR-specific work, similarly stressed that resistance is fundamentally driven by human activities, including farming and chemical use in the broader environment. Three respondents pointed out the role of farmers in AMR: *“Most of [the farmers] are in the bushes. And they are always near the water catchment area […]. Because the animals […] come and drink water*,* so they come*,* they defecate. And these bacteria*,* because of the numerous antibiotics that they give to the animals*,* maybe the bacteria in them will become resistant. They enter the water source” (institutional actor*,* male*,* 41–51 years*,* environmental health*,* practitioner*,* regional level)*.

Respondents from the human health sector emphasised self-medication, “abuse”, “inappropriate” and “indiscriminate” use of antibiotics by community members and farmers, low awareness of AMR, and underdosing in both humans and animals as key contributors to resistance. They also highlighted the use of chemicals in crop production and the limited availability of a wide range of antibiotics, which they felt created selective pressure and constrained rational prescribing.

### AMR as a structural and economic problem

Alongside individual-level explanations, participants, particularly institutional actors and farmers, framed AMR as embedded in wider structural and economic conditions that shape antibiotic use and its consequences. A few farmers pointed to high drug and feed prices and limited government support as key constraints influencing how they used antibiotics, without explicitly connecting them to AMR (field diary, Agogo, 29 April 2022). Three institutional animal health professionals emphasised that AMR impacts not only human health but also has economic implications for the livestock industry, including work productivity, employment, income, and production capacity and costs.

Environmental health framed AMR as a product of human activities that intersect with environmental management and agricultural systems: *“All human activities*,* all food chain activities end up impacting the environment” (institutional actor*,* female*,* 41–51 years*,* environmental health*,* research*,* national level)*. However, one institutional actor noted that awareness of AMR among environmental health actors remains limited, potentially constraining cross-sectoral responses: *“The issue is that a lot of people even with the agency itself*,* the staff*,* the officers*,* when it comes to AMR in the environment*,* we really do not understand” (institutional actor*,* female*,* > 52 years*,* environmental health*,* policy*,* national level)*.

Human health professionals highlighted poor infection prevention and control in health facilities as a critical structural driver of AMR. Two respondents described how prolonged illness periods attributed to resistance created economic burdens, both through lost income and the demands of caring for sick family members which results in *“losing your salary or your productive work time that you could have been using for something else” (institutional actor*,* female*,* 41–51 years*,* human health*,* policy*,* national level)*. Some questioned the relative value of community-level awareness-raising compared with more systemic interventions targeting antibiotic practices and stewardship within hospitals: *“I don’t know if the key for AMR is awareness of a woman in a community in a village […] [and] how much that is important compared to all hospitals in the world massively giving antibiotics to their patients” (institutional actor*,* male*,* > 52 years*,* human health*,* policy*,* national level)*.

### Everyday exposure and interconnected animal-environment-human relations

Several community members reported that they could not assess the presence of AMR in animals or comment on possible links between animal and human health due to little or no experience with animal rearing. Others expressed scepticism, stating that they did not believe diseases could be transmitted from animals to humans. However, a few community members recognised connections between human health, animals and the environment. Three respondents emphasised the role of environmental cleanliness and close contact with animals in shaping disease risks: *“I think diseases that spread from animals to humans are mostly caused by dirt. When you do not keep the surroundings clean. When you personally take care of a sick animal who defecates and urinates*,* you can pick it up because you will be the one to sweep the dump” (community member*,* female*,* 30–40 years*,* trader)*. Another respondent discussed how environmental conditions, food sources, and disease vectors may influence antimicrobial resistance: *“It may be where you got the sickness from*,* that is contributing to the antimicrobial resistance because maybe you got the sickness from where you stay or from what you eat. […]. You can even contract a disease by eating meat. […] It could be unhygienic environment at where one stays. […]. Animals such as mosquitoes and houseflies can cause that because they can transfer it to you through their bites” (community member*,* 30–40 years*,* crop farmer)*.

Many farmers recognised that livestock management practices, hygiene, and antibiotic use can have consequences that extend beyond animal health. Most respondents recognised a clear link between animal and human health, citing the transmission of diseases between animals and humans through close contact or the consumption of animal products (field diary, Agogo, 15 June 2022). However, one farmer expressed scepticism, stating that they did not believe diseases could be transmitted through direct contact between livestock and humans. One respondent noted that safe cooking practices can help prevent such transmission, while another explained: *“If you give it [a pharmaceutical] to the animal and it has become a lot in their system [body]*,* if humans eat it*,* it has an effect on them” (farmer*,* female*,* 41–51 years*,* co-owner with her husband of a semi-intensive poultry farm)*. Similarly, one farmer emphasised the role of environmental conditions in livestock health: *“The first thing I will check is where I rear them. The environment*,* there may be an animal [organism] worrying them. […] because my colleague’s animals are growing fine*,* whilst mine are not*,* then I need to check that first” (farmer*,* male*,* 30–40 years*,* cattle backyard farming)*.

When we asked a question related to the antibiotic withdrawal period, thus not selling eggs or slaughtering livestock for several days after the administration of antibiotics, several respondents mentioned the importance of observing it as *“[t]he drug will still be in the meat*,* so if you consume it*,* you can get an effect” (farmer*,* male*,* 18–29 years*,* commercial mixed farm)*. While farmers demonstrated awareness of potential risks to human health, they described adhering to the withdrawal period as challenging or even impossible due to financial losses, for example due to required discarding of eggs, as they noted during unstructured interviews. A few farmers explicitly recognised that AMR and other illnesses can be transmitted between humans and animals, linking their own practices to wider patterns of cross-species disease risk: *“Yes*,* I see it [AMR] as […] [an] illness [as well] and it is possible. It can be transferred from human to animal and can also be transferred from animals to human” (farmer*,* female*,* > 52 years old*,* small-scale pig farm and household poultry farm)*.

Beyond these concrete concerns about contact and consumption, some community members and farmers articulated broader understandings of animal-environment-human interdependence, while institutional actors more consistently framed One Health in systemic terms. Community respondents implicitly evoked such interdependence through references to environmental cleanliness, food sources, and vectors, while farmers who linked livestock management and environmental conditions to animal health extended this logic to human health. Institutional actors, by contrast, framed One Health in more consistently systemic terms.

### Weak One Health implementation and cross-sectoral imbalance

Although awareness of One Health as a concept was generally high among institutional actors, respondents frequently highlighted gaps and imbalances in its practical implementation. One animal health professional emphasised that while understanding of One Health extends from the district level to national policy-makers, efforts to translate this into coordinated action remain limited in practice. However, participants provided little detail on what effective implementation would look like in their context. Another institutional actor offered a more optimistic view, stating that *“so far*,* so good. We are on course*,* and it’s been done in the spirit of One Health*,* with the environment*,* there is animals that are there*,* there is humans” (institutional actor*,* male*,* 30–40 years*,* animal health*,* policy*,* national level)*. A further respondent critiqued the lack of concerted efforts specifically in the field of AMR, suggesting that One Health is often invoked rhetorically rather than operationalised: *“From the animal health point of view*,* we are doing very [little] when it comes to AMR. So*,* we don’t generate enough data. […] So*,* all that we are seeing about AMR is snippets of works that people are doing. We don’t all have a concerted effort with a common goal that is going to really change policy. Everybody is doing something in their corner” (institutional actor*,* male*,* 41–51 years*,* animal health*,* practitioner*,* regional level)*.

Environmental health professionals described feeling underrepresented in national and international AMR efforts and saw this marginalisation as a barrier to genuine One Health collaboration. *“[T]he only imbalance I see is that the health ministry*,* they have a lot of activities on AMR so that is their strength. Because they started*,* I think a lot of funding agencies and donors tend to link towards the health sector […]. But recently*,* with FAO [Food and Agriculture Organization] also coming in*,* I think the animal and the plants are also doing better than the environment. As for the environment*,* we are just far behind” (institutional actor*,* female*,* > 52 years*,* environmental health*,* policy*,* national level)*. Several human health respondents explicitly linked the complexity of AMR to the need for cross-sectoral collaboration, while acknowledging the challenges of its implementation: *“[E]ven at a district level we are not working as a unit. The health system is working on its own*,* environment and agric[ulture] as well […]. So even at this small level we are not working together*,* that is a problem. So*,* how do you expect it to even work at the regional or national level?” (institutional actor*,* male*,* 41–51 years*,* human health*,* practitioner district level)*.

## Discussion

This study explored how community members, farmers, and institutional actors in Ghana’s Asante Akim North Municipality understand AMR and the interconnections between human, animal, and environmental health. Drawing on anthropological and socio-epidemiological fieldwork, we found a striking divergence: AMR was largely absent from the lived experience of community members and farmers, while institutional actors framed it as an urgent crisis. This gap shapes how each group makes sense of antibiotic ineffectiveness and One Health in the context of AMR.

Perceptions of AMR differed significantly across stakeholders. Most community members and farmers never heard of AMR, echoing findings from previous studies across diverse settings and societal strata [24–27]. Despite receiving information about AMR, many community members questioned whether AMR exists, especially in the absence of personal experience. Others appeared unconcerned, viewing occasional antibiotic ineffectiveness in human or animal health as a normal, individual response rather than a broader collective health threat. While several farmers acknowledged that AMR could be transferred like a disease, they did not perceive it as a pressing issue, as they had not yet experienced negative impacts themselves.

Community members and farmers seemed to be accustomed to pharmaceuticals, including antibiotics, “not working”, but they typically did not associate this phenomenon with AMR, interpreting it instead as part of everyday illness and experiences of pharmaceutical use. This aligns with prior research showing that community members experience some antibiotics as ineffective but treat this as an individual problem rather than linking it to the broader issue of AMR [28]. In contrast, institutional actors consistently framed AMR as a high-priority, urgent challenge, closely aligning, with the global discourse that identifies it among the top ten threats to global health [11, 29]. However, only few institutional actors had personal or direct professional experience with AMR. Their framing of AMR seemed to be less experiential and more influenced by exposure to global AMR discourse, the national AMR policy context, and donor-funded programmes. In this analysis, global AMR discourse is understood as encompassing two key sources. The first is the formal education that institutional actors received in university or specific workshops. The second is the internationally circulating narratives, agendas, and policy frameworks promoted in official documents such as the One Health Joint Plan of Action by the Quadripartite or the Global Action Plan on Antimicrobial Resistance by the WHO, which characterise AMR as a ‘silent pandemic’ driven in part by antibiotic misuse among community members and farmers [30, 31]. This influence appeared to be reflected in institutional actors’ language during the interviews, when they used similar terms and emphases. It is unsurprising that institutional actors are highly aware of AMR, as they are actively targeted and involved in transnational knowledge flows through participation in international conferences, collaboration with technical and financial partners, and engagement in AMR-related research and interventions, suggesting that these knowledge flows provide the interpretive lens through which they understand and communicate AMR.

Community members, farmers, and institutional actors drew on diverse explanatory models to make sense of antibiotic ineffectiveness and AMR. While institutional actors largely framed AMR within biomedical concepts, such as antibiotic overuse, underdosing, and poor regulation, community members and farmers explained AMR through experiential models. These contrasting knowledge forms highlight different pathways through which understanding of AMR is constructed. Our finding that both community members and farmers often attributed AMR to individual physiological differences, such as the incompatibility of blood and drug, habituation to drugs, or the strength of certain illnesses overruling weaker medications, is consistent with findings from other LMICs [32–34]. These experiential explanations ranged from bodily incompatibilities to habituation to the perceived strength of illness. For instance, some participants in our study believed that a drug’s effectiveness depends on compatibility with one’s blood type, illustrated by malaria medication failing to work for certain individuals. Others perceived that drugs like paracetamol lose efficacy after frequent use, and that particularly strong illnesses simply overrule weaker medications. These logics reflect a broader perception documented in the literature that pharmaceuticals are personalised, whereby a drug’s effectiveness depends on its compatibility with individual characteristics rather than its intrinsic properties alone [35–37]. Treatment failure is thus attributed to individual factors rather than to the drug itself or, in the case of antibiotics, to AMR. Echoing our findings, previous studies among community members and farmers, including in the so-called Global North, have documented persistent misconceptions, such as the belief that AMR only affects those with frequent antibiotic use and that it reflects the body’s resistance [38–42]. While these explanations diverge from biomedical understandings of AMR, they nonetheless represent coherent local logics that integrate personal experience and observed patterns of treatment failure.

Farmers, by contrast, drew on a hybrid explanatory framework that combined experiential knowledge with elements of the biomedical language used by institutional actors. Like community members, they linked AMR to repeated use of antibiotics and emphasised environmental factors, such as unsanitary conditions, as contributing causes. At the same time, many farmers adopted terminology attributing AMR to ‘overuse’ or ‘abuse’ of antibiotics, language that closely mirrors the messaging of awareness campaigns and educational programmes. This discursive alignment may reflect the influence of information disseminated by institutional actors on how farmers talk about AMR, while they are simultaneously exposed to competing messages and incentives within the food production system. Farmers have historically been actively encouraged by governments, pharmaceutical companies, and agricultural authorities to use antibiotics for growth promotion, prophylaxis, and disease prevention [43].

In this context, farmers’ responses may partly reflect what they perceive as expected or socially desirable answers, as they negotiate these contradictory sources of knowledge and competing demands, rather than internalised views. However, this discourse does not necessarily translate into practice. Farmers’ antibiotic use remains strongly shaped by structural constraints, including limited access to veterinary services and economic pressure to avoid production losses [27, 44, 45]. For many, this is less about seeking profit and more about securing a basic income for survival, producing a tension between awareness of recommended practices and the realities of daily life. For instance, while most farmers in our study demonstrated good awareness of the antibiotic withdrawal period and its importance in preventing residues in food, they also admitted struggling to comply. Similar findings from Kenya and Tanzania show that, despite high awareness, implementation of the withdrawal period is often hindered by potential income loss and weak monitoring systems [46, 47]. Other studies similarly show that awareness does not automatically translate into compliance or behavioural change, particularly when interventions impose costs that are not offset by adequate institutional support [27, 48, 49].

Institutional actors across all sectors predominantly adopted a biomedical and policy-oriented framing of AMR. While they understood AMR as a complex, multifactorial issue, their narratives often placed primary responsibility on community members and farmers, echoing the dominant global framing of AMR as a behavioural problem at individual level [39, 50]. Only one institutional actor partially challenged this narrative by questioning whether focusing on community-level awareness was sufficient, given the widespread and frequent prescription of antibiotics in hospitals. While this position shifted attention away from patients, it continued to locate responsibility at the individual level by attributing blame to healthcare professionals rather than to systemic factors influencing prescribing practices. This tension reflects a broader critique in the literature: global AMR strategies disproportionately emphasise local behaviours while neglecting structural and regulatory factors of antibiotic use and systemic drivers, such as the simultaneous difficulty of accessing public healthcare services and the easy availability of antibiotics through unregulated private providers [12, 49, 51, 52].

Across these three explanatory frameworks, experiential, hybrid, and biomedical, AMR is framed as an individual problem located within the body, attributed to personal behaviour, or blamed on individual practices. This individualising framing carries consequences at both community and institutional levels. At the community level, institutional expectations of behaviour change are misaligned with community members’ perceived external locus of control, the tendency to attribute treatment outcomes to forces beyond their personal influence, such as drug strength, fate, or individual physiology. At the institutional level, the same individualising focus shifts attention toward personal responsibility while obscuring the concept of transmission of resistant bacteria across sectors, potentially fostering a sense that the problem cannot be explained or acted upon collectively [53].

Institutional actors in our study endorsed the One Health concept as essential for tackling the multifactorial challenge posed by AMR holistically, aligning with the global policy discourse [30, 54]. However, their accounts pointed to a disjuncture between this rhetorical consensus and the fragmented, uneven ways in which it is currently enacted on the ground. Although national AMR strategies, including Ghana’s, are formally grounded in One Health [15], the human health sector continues to dominate AMR agendas, while animal health receives comparatively less attention and environmental health remains consistently underrepresented and underfunded [55]. This marginalisation was reinforced by interview accounts that framed the environment as a passive sink for contamination from animal and human health sectors rather than as an active domain shaping AMR emergence and spread. Interview respondents from the animal and environmental health sectors themselves frequently reinforced a human health focus, fostering anthropocentric views that hinder truly interdisciplinary collaboration in line with One Health principles. As a result, data availability and integration remain limited and cross-sector collaboration weak, echoing findings from other settings [16, 56, 57].

Moreover, Ghana’s national One Health architecture, including a National Action Plan on AMR, a One Health technical working group, and an AMR platform, has not translated into equally strong district-level implementation [16]. Contributions from institutional actors across all sectors were often theoretical and lacked concrete examples of integrated practice, a gap particularly pronounced in the environmental health sector, whose chronic underfunding and absence from AMR initiatives mirrors international trends [58].

Yet, alongside this institutional picture, community members and farmers offered experience-based insights that resonate with One Health principles, even if the term was not mentioned during the interviews. This mirrors findings from a study among the general public in Beijing, China, where awareness of the term ‘One Health’ was low despite high recognition of its underlying principles [59]. While not all community members knew about the interconnection between animal and human health, some provided rich, experience-based explanations linking disease transmission to meat consumption, unclean environments, waste exposure, and insect vectors. Farmers described cross-species disease risks grounded in their daily work, for instance through contact with or consumption of animal products. Awareness of zoonotic transmission pathways among livestock farmers has been shown to vary considerably across contexts, shaped by occupational exposure and production systems [60, 61]. In our study, such knowledge may represent an intuitive, tacit understanding emerging from lived experience rather than from theoretical scientific knowledge [62]. Yet, as others have noted, farmers’ experiential perspectives often remain overlooked in policy and intervention design [63].

These findings raise the question of what a genuinely inclusive One Health approach might look like if it took community and farmer knowledge as a starting point. As anthropological work by P. Descola [64] and F. Keck [65] has suggested, local understandings of animal-environment-human relations can challenge biomedical assumptions and enrich One Health by foregrounding multispecies relationships and context-specific knowledge. Whether and how such experiential insights could productively interact with, and potentially transform, institutional One Health frameworks remains an open question.

This study has several limitations. Participant categories, community members, farmers, and institutional actors, were defined analytically to capture distinct relationships with antibiotic use and AMR. We acknowledge, however, that these categories overlap in practice: farmers are also community members, and district-level institutional actors may reside in the communities studied. Nonetheless, the findings validate this classification, as farmers demonstrated knowledge patterns distinct from other groups by virtue of their occupation, and institutional actors similarly expressed unique, sector-specific perspectives. This supports the analytical rationale underpinning the categorisation.

Institutional actors were purposively selected based on their professional involvement in AMR-related management, regulation, or research. This group therefore cannot be considered neutral with respect to AMR awareness. Their responses reflect informed, sector-specific perspectives rather than general institutional opinion. While this was a deliberate sampling strategy to capture expert and policy-relevant viewpoints that complement community-level data, findings concerning institutional AMR perceptions should be interpreted with this caveat in mind. A further limitation is that the relatively small number of institutional actors precluded comparison across local, regional, and national levels. Moreover, the single-district focus means that our findings may not be representative of the national situation in Ghana. Social desirability may have led some respondents to reproduce official discourses, indicating honesty in reporting learned knowledge about AMR. However, because they were essentially repeating memorised content, we did not capture what they personally thought or how they critically engaged with the issue beyond the official narrative.

This study also has several strengths. The interdisciplinary approach, integrating anthropology and socio-epidemiology, allowed for triangulation across methods, data sources, and investigators, lending the analysis both depth and interpretive rigour. To our knowledge, this is the first study to examine and compare understandings of AMR and One Health in the context of AMR across this wide range of stakeholders, from rural community members and poultry farmers to institutional actors from animal, environmental, and human health sectors at district, regional, and national levels.

## Conclusions

This study demonstrates that understandings of AMR diverge across stakeholder groups. While community members and farmers drew on experiential logics to explain AMR and the interconnectedness of animal, environmental, and human health, institutional actors reproduced a global framing centred on individual responsibility, a framing that may be shaped by their engagement with policy networks, global discourses, and funding mechanisms. These contrasting explanatory models have tangible consequences for AMR interventions. Initiatives that rely too heavily on global biomedical models, risk failing to address local realities as they rest on assumptions about how AMR is understood that may not align with the experiential logics held by community members and farmers. Effective interventions must therefore integrate pluralistic explanatory models, ensuring that experiential and biomedical knowledge are equally valued, and involve diverse stakeholders across disciplines and backgrounds throughout intervention design and implementation. Our findings further revealed that community members and farmers already draw on lived experience that embodies One Health principles in ways that institutional actors, despite rhetorical commitment, have yet to fully operationalise. We hypothesise that integrating such experiential knowledge could enrich One Health-based AMR interventions, and we propose that future research test this proposition through participatory approaches that treat communities and farmers as valued knowledge holders rather than simply as targets of awareness campaigns. Such an approach will be essential if One Health is to move from a policy paradigm to effective, contextually appropriate, and sustainable practice.

## Supplementary Information

Below is the link to the electronic supplementary material.

Supplementary Material 1: COREQ Checklist.

Supplementary Material 2: Table S1.

Supplementary Material 3: Guides and information sheets.

## Data Availability

Interview guides and information sheets from anthropology and socio-epidemiology and the observation guides (anthropology), are provided in Supplementary Material 3: Guides and information sheet. The full interview transcripts are not publicly available due to participant confidentiality concerns, as they contain detailed narratives that could potentially identify participants despite anonymisation, which conflicts with the confidentiality assurances given during informed consent. These transcripts are available from the corresponding author (maresa.neuerer@uni-heidelberg.de) on reasonable request, subject to a data-sharing agreement that safeguards participant privacy and aligns with the ethical approvals obtained for this study.

## Abbreviations

AMR: Antimicrobial resistance
IDIs: In-depth interviews
KIIs: Key informant interviews
LMICs: Low- and middle-income countries
