# Supplementary material for: Divergent stakeholder perspectives on antimicrobial resistance and One Health in Ghana: a qualitative study
Source: BMC Glob Public Health. 2026 Aug 19;4:82. doi: 10.1186/s44263-026-00319-1 (PMC13491848; doi:10.1186/s44263-026-00319-1)
Supplement: Supplementary file 2 [file 44263_2026_319_MOESM2_ESM.pdf]

Table S1: *Demographic and key characteristics of the final analytical sample by discipline and interview type*

Discipline	Interview type	Participants	No. (total)	Gender		Age range (yrs)	Experience range (yrs)	Key characteristics
				M	F			
**Anthropology**	In-depth interviews	Community members	45	13	32	21-75	-	**Setting:** rural (n=21), semi-urban (n=24). **Socio-economic status:** lower class (n=13), middle class (n=20), upper class (n=12).
		Farmers	14 (from 6 farms)	11	3	18-69	-	**No. of birds:** 500-1000 (n=1), 1000-5000 (n=3), >5000 (n=2). **Date of opening:** before 2010 (n=2), 2010-20 (n=2), after 2020 (n=2).
**Socio-epidemiology**	Key-informant interviews	Institutional actors	25	19	6	-	1-33	**Sector:** animal health (n=9), environmental health (n=6), human health (n=10). **Level:** district (n=6), regional (n=9), national (n=10).
	In-depth interviews	Community members	11	4	7	18-72	-	**Recruitment place:** chemical shop (n=5), pharmacy (n=5), community health centre (n=1).
		Farmers	8	7	1	23-64	-	**Recruitment place:** veterinary shop (n=7), informal veterinary market (n=1).
