# Supplementary material for: Divergent stakeholder perspectives on antimicrobial resistance and One Health in Ghana: a qualitative study
Source: BMC Glob Public Health. 2026 Aug 19;4:82. doi: 10.1186/s44263-026-00319-1 (PMC13491848; doi:10.1186/s44263-026-00319-1)
Supplement: Supplementary file 3 [file 44263_2026_319_MOESM3_ESM.pdf]

ANTHROPOLOGY – OBSERVATION GUIDES

Observation guide and open discussion with households in the villages for human health and animal health

These observations will be conducted by the junior researcher in areas of Agogo and in the village of Bebome, where he will be housed.

As many families/households as possible will be observed on these topics. During the observations, open discussions will be held with the people present on the topics of interest in the study. The word "antibiotic" should not be mentioned directly. It is important to be able to understand what people think about the different molecules that we know, us, as being antibiotic and to understand for which ones they may use the word "antibiotic" and for which ones not.

Written consent will be obtained beforehand, with the assembly man of the areas and village (see information and consent forms).

1) Human health

- What are the different drug distributors (public, private, informal) for human health in the villages studied or nearby?

- What are the different health centers in or near the villages studied?

- What are the hygiene practices in the family? Are they facilitated or not?

- What are the self-medication practices (either from the person alone, or from his family environment, friendship environment) in the households and what meaning do people give to these practices for children, for adults, for the elderly?

- Who are the people with whom health issues are discussed? the decisions taken?

- Are antibiotics involved in these self-medication practices, which ones (molecules, marketing name, country of manufacture, producers, wholesalers, packaging) and for what health issues? Where are they purchased and at what price? Who paid and how was the money found? How are they taken and in what dosage?

- Do households have home pharmacies and if so, what are their contents? If so, a description should be provided.

- What are the consultation practices of the households, with which health centers/health professionals (biomedical, such as "traditional," religious, spiritual, informal) and for which health issues for children, for adults, for the elderly?

- Are antibiotics involved in these prescriptions, which ones (molecules, marketing name, country of manufacture, producers, wholesalers, packaging) and for which health issues? Where are they purchased after the prescription and at what price? Who paid and how was the money found? How are they taken and according to what dosage?

- Are alternative phytomedicine products to antibiotics used in self-medication or following a prescription? If so, which ones (molecules, marketing name, country of manufacture,

producers, wholesalers, packaging) and for what health issues? Where are they purchased and at what price? How are they taken and in what dosage?

- Are there differences in care depending on the members of the family (children, adults, working men, working women, pregnant women, elderly etc.)?

- What are people's perceptions of the different antibiotics they encounter?

- How are expired medications or medications that people want to get rid of managed? Where are they disposed of and how?

- Where do people relieve themselves? Is it in nature or in a bathroom?

- Do people know about antibiotic resistance as it relates to human health? If so, what do they know about it and how did they learn about it?

2) Animal health

- What are the different drug distributors (public, private, informal) for animal health in the villages studied or nearby?

- Do all families/households in the villages raise food crops, or do only some people do so?

- What types of animals are raised in this way?

- What are the hygiene practices in the family around animals? Are they facilitated or not?

- Are medicines given to these animals for productivity or in relation to animal health issues? If so, which ones? Are there antibiotics among them and if so which ones (molecules, marketing name, country of manufacture, producers, wholesalers, packaging) and for what health issues? Where are they purchased and at what price? How are they given and in what dosage?

- Do people have pharmacies for the health of their animals and if so, what is their composition? If so, a description should be given.

- Are alternative phytomedicine products to antibiotics used for the animals raised? If yes, which ones (molecules, marketing name, country of manufacture, producers, wholesalers, packaging) and for what health issues? Where are they purchased and at what price? How are they given and in what dosages?

- Do people use animal health professionals and if so which ones? Do these professionals travel or do people go to them? If they travel, how are the consultations conducted? Do these professionals recommend antibiotics? If yes, which ones, where do they buy them, at what price? How are they given to animals and in what dosage?

- What are people's perceptions of the different antibiotics encountered in animal health?

- How are outdated medications or medications that people want to get rid of managed? Where are they disposed of and how?

- Do people have any knowledge about antibiotic resistance in relation to animal health? If so, what do they know and how did they learn about it?

3) The overall understanding of the village

- Roughly in the middle of the ethnography, try to draw a diagram of the village precisely and the issues in the village (related to health or not, indicate the places where for the people and animals to wash, waste management, places of power, places of worship, tontine / microcredit groups, places of distribution of products, market, circulation of water, circulation of sellers ...) with people with which the researcher interacted the most with. And take a picture of this diagram.

Observation guide and open discussion in intensive or semi-intensive livestock farms

These observations will be conducted by the junior researcher and in Ghana by Carine Baxerres and the post-doctoral fellow at a few poultry farms for several weeks each.

During the observations, open discussions will be held on the topics of interest of the study with the individuals present. The word "antibiotic" should not be mentioned directly. It is important to be able to understand what workers think about the different molecules that we know, us, as being antibiotic and to understand for which ones they may use the word "antibiotic" and for which ones not.

Written consent will be obtained beforehand, with the owner of the farms (see information and consent forms).

1) Location and context of the farm:

- Describe the farm in great detail.

- Make a precise diagram of the place and take a picture of it. Possibility of doing it with people who work in the farm.

- What are the hygiene practices in the farm? Are they facilitated or not?

- Describe its environment.

- What are the sources of information and how does information circulate, particularly on antibiotics?

2) Operation of the farm:

- Describe precisely the different activities carried out on the farm.

- Describe precisely the rhythm and the way of life of the animals.

- Describe precisely the different tasks carried out by the farm personnel, and in particular the activities related to the use of medication and/or phytotherapy products for the production or health of the animals.

- Describe the relationships among the farm's staff and with its managers.

- Describe the farm's relationship with animal health professionals and the specific interactions, if any. Who in the farm interacts with these professionals?

- Describe the links between the farm and regulatory actors, if there are any? Who in the farm interacts with regulatory actors? How is it going (inspections, checks, etc.)

- How are the prices of animals regulated? Who influences this? How are pricing decisions made?

- Describe the farm's waste management.

3) Medications/Products Used:

- What drugs/products / other inputs (food, equipment, animals, eggs, seeds) are used for production or animal health and in what ways and dosages?

- Which antibiotics or alternatives to antibiotics are used (molecules, marketing names)? Make an inventory.

- In what forms are antibiotics or alternatives to antibiotics used? (tablets, bulk, blister packs, syrups, injectable ampoules, etc.)

- What is the content and form of the presentations and packaging of antibiotics or alternatives to antibiotics?

- What are the countries of manufacture of the antibiotics or alternatives to antibiotics, their producers and from which wholesalers are they purchased?

- How are the drugs/products procured (where, by what processes, by whom, etc.)?

- Discuss with the farm staff the expected effects (diseases, symptoms treated, side effects) of antibiotics or alternatives to antibiotics.

- Do farm staff have any knowledge of antibiotic resistance as it relates to human or animal health? If so, what do they know about it and how did they learn about it?

- How are used medications/products / other inputs stored (wholesale, retail, etc.) and how are expired medications managed? Where and how are they disposed of/transferred elsewhere?

5) Relationships between the farm and its customers:

- How is the sale conducted? (duration of the interaction, location, advice given, networking, customer requirements)

- How are the prices determined? Quality elements?

- Who are the customers? Where are they (region, capital, abroad, products for local, national consumption, for export) (the possibility of conducting free interviews with customers will be questioned during the study, according to the empirico-inductive approach of anthropology)

- How are the animals (dead or alive) transported?

ANTHROPOLOGY – INTERVIEW GUIDES

Interview guide in villages for human and animal health

These interviews will be conducted by the junior researcher during the second half of his immersion in the areas and village. They will be conducted with 30 families: the mother, and sometimes – when relevant – the father and/or a grand-mother.

Written consent will be obtained prior to the interview (see information and consent forms).

Interview Part 1: Human Health

Starting question: To begin, I would like you to tell me how you deal with health issues in your household, your own, your children's, your spouse's (if any). What health problems do you have most often? What do you do initially when it starts? What do you do afterwards if it continues? What products do you use? What professionals might you see? What else might you be doing to stay healthy or not get sick? I'm going to take the first name of each member of the household (people eating from the same pot) and you're going to tell me about the last time each of you had to do something related to health (take a product, go to a doctor, go to a traditional therapist, maybe pray too, etc.), maybe for an illness or more generally to maintain good health, maybe after having gone to a doctor or on your own initiative or under the advice of someone you know So let's start with...

1) Daily health management

- What are their preventive, curative and health maintenance self-medication practices? With what types of products (pharmaceutical drugs, standardized and non-standardized herbal products, others), purchased where, at what price, prepared by whom, found where, advised by whom, dosages of use?

- What are the health consultation practices? What types of professionals (biomedical (Public or private), "traditional", religious, other), chosen how, at what price, what prescriptions, bought where, at what price?

- To be told about the therapeutic itineraries and the meaning put behind these itineraries.

- Does he/she ever throw away medicines? In what cases? Where and how?

2) Uses and perceptions of antibiotics

- Go back to the different antibiotics mentioned and try to find out for what health issues this product is used, how it is taken (dosage), can all members of the household use it?

- Where are these products purchased, how much are they purchased for, why are they purchased there?

- What is the difference between these different products?

- What does he/she think about antibiotics in general (effectiveness, old and new products, side effects, for what health issues)? How does he/she know all this about antibiotics?

- Does he/she use herbal medicine (standardized or not) as an alternative to antibiotics? Who advised him/her? Where does he/she buy it?

- *Did the issues of antibiotic resistance come up in the previous discussion and if not,* is he/she aware of this issue *(to be formulated so that the person understands without using this term)*, if so where did he/she learn about it, what does he/she think about it?

3) Discussion about the home pharmacy (This should be done after the interview). Information derived should be written in the word file after the interview.

- *Does he/she have a home pharmacy and if so,* is it possible to see it and discuss together about the products it contains? (take one by one the different products and have them tell you why it was bought, where, how much, why can it be used too, etc.).

If possible, take a picture of the home pharmacy and where it is stored. List (on a sheet/book) the medicines in the home pharmacy, note the molecules, marketing names, countries of manufacture, producers, wholesalers, describe the packaging; and extend the interview discussions by asking about the usefulness of certain medicines and/or why they were used.

Observe how medicines are stored, what the home medicine cabinet looks like, where it is stored, whether there are several in the family, how medicines are kept, what they look like (dirty, damaged...), etc.

Part 2 of the interview: Animal health (for those who practice food production)

Starting question: To continue, I would like you to tell me about the way you take care of the animals you raise, especially their health issues? What animals do you raise? How do you handle them in general? What about their health? Do you give them products regularly? Do you ever consult with animal health professionals?

1) Animal management and health

- What animals does he/she raise and for what purposes (household consumption, sale, savings, etc.)?

- How does he/she take care of them in general? Can he/she describe a typical day of management of the different animals?

- Does he/she use medication or other products to take care of his/her animals? Which ones (molecules and composition)? For what purposes (production, health, curative, preventive, etc.)? Where are they purchased, how much, from whom, why there?

- How did he/she learn to use these products? Did he/she attend any training? Has he/she received advice and from whom?

- Does he/she receive visits from animal professionals? If yes, which ones and why?

- Does he/she ever go to see animal professionals? If yes, which ones, for what health issues and how? Do they prescribe products? If yes, which ones and where does he/she buy them?

- How does he/she manage the waste from his/her livestock (feces, leftover feed, leftover products)? What do they do with it?

2) Uses and perceptions of antibiotics

- Go back to the different antibiotics mentioned and try to find out for what health issues these products are given to the animals? or ask if he/she uses antibiotics for his/her animals? If so, which ones and how are they given (dosage)?

- Where are these products bought, how much are they bought for, why are they bought there?

- What is the difference between these different products?

- What does he/she think about antibiotics in general for animals (effectiveness, old and new products, side effects, for what health issues)? How does he/she know all this about antibiotics for animals?

- Does he/she use herbal medicine (standardized or not) as an alternative to antibiotics for his/her animals? Who advised him/her? Where does he/she buy it?

- What does he/she do with the antibiotics bought for the animals that are outdated or that he/she no longer wants? how does he/she dispose of them?

- *Did the issues of antibiotic resistance come up in the previous discussion and if not,* is he/she aware of this issue *(to be phrased in a way that the person understands without using this term)*, if so where did he/she learn about it, what does he/she think about it?

3) Discussion about the farm animal pharmacy (This should be done after the interview)

- *Does he/she have a pharmacy for animals and if so,* is it possible to see it and discuss together about the products it contains? (take one by one the different products and have the person tell why it was bought, where, at what price, why can it be used too, etc.)

If possible, take a picture of the pharmacy and where it is stored. List (on a sheet/book) the medicines in the pharmacy, note the molecules, the marketing names, the countries of manufacture, the producers, the wholesalers, describe the packaging; and extend the discussion from the interview.

Observe how the medicines are stored, what the home pharmacy looks like, where it is stored, whether there are several in the family, how the medicines are kept, what they look like (dirty, damaged...), etc.

Characteristics of the respondent *(to be filled in at the end of the interview, once the recorder is turned off, for information that will not be revealed during the interview)*

Level of education :

Profession/activities:

Age:

Renter/Lives in a relative's house/Owner:

Average monthly income: (from Observation, try to put the person in a category ‘rich’, ‘middle class’, ‘poor’)

Average monthly household income:

Mother tongue/sociolinguistic group/Geographic origin (where was he/she born):

Religion, if Christian specify church:

Owns a vehicle?, if yes, which one(s):

Note the socio-economic level of the household after the interview is conducted and the home is observed ("affluent," "intermediate," "impoverished")

Interview guide in intensive or semi-intensive livestock farms

These interviews will be conducted by the junior researcher, the post-doctoral fellow and Carine Baxerres, during the second half of the ethnographies of the farms. They will be conducted with the staff of the farms: employees and/or owners. The word "antibiotic" should not be mentioned directly. It is important to be able to understand what people think about the different molecules that we know, us, as being antibiotic and to understand for which ones they may use the word "antibiotic" and for which ones not.

Written consent will be obtained beforehand (see information and consent forms).

Starting question: To begin with, I would like you to tell me about your professional career up to now and this farm in which you work. Did you have an education? What other jobs have you had before? How did you get started here?

1) Professional background

- Schooling, secondary studies, continuing education

- the different activities and jobs occupied

2) Work on the farm

Can you tell me about the farm where you work? What is its history? How did it evolve? Can you also tell me how you manage the farm? How do you do it? What are the tasks you have to perform?

- History of the farm and its evolution over time. Is it linked to a larger structure? Where does the capital come from? Are there shareholders? Where do the animals come from initially?

- History of semi-intensive poultry farming, and other livestock farming in general (pigs, sheep, cattle, etc.), in Agogo. How long have semi-intensive farms been in existence, how have they developed over time, is it a successful and growing sector, and so on.

- Tasks performed by the respondent.

- Where does the feed for the animals come from? What other inputs are used on the farm?

- Difficulties encountered.

- Relationships among farm personnel.

- Strategies for developing the productivity of the farm. If products are used, ask which ones, why, and where they were purchased. Insist particularly if antibiotics are used (molecules, commercial names, prices).

- Who are the customers and how are they sold?

- How is the waste from the farm managed?

3) Animal health management

- What animal health problems are encountered? How do they deal with them?

- Do they use medication or other products to prevent and treat animal diseases? Which ones, bought where, at what price? How do they know about these products? How do they give them to the animals (dosages, method of administration)? Do people come by to promote and sell these products to the farm?

- Do they use animal health professionals? In what cases? To whom? Do they travel to them or do they come? Where are they based? How much does it cost? What do they advise them to use in terms of medicines or other products? Where do they buy these products? How much do they charge?

- What do they do with the medicines or other products they use? Where are they stored? Who manages this place? What happens to products that are out of date or that they want to get rid of?

4) Uses and perceptions of antibiotics

- Go back to the different antibiotics mentioned and try to find out for what health issues these products are given to animals? or ask if they use antibiotics for animals? If so, which ones and how are they given (dosage)?

- Where are these products bought, how much are they bought for, why are they bought there?

- The history of the veterinary pharmaceutical market in the region. Where were veterinary pharmaceutical purchased when the farm first started, and where have they been purchased since then up to the present day? From which suppliers have they been able to purchase these products since the beginning?

- What is the difference between these different products?

- What does he think about antibiotics in general for animals (effectiveness, old and new products, side effects, for what health issues)? How does he/she know all this about antibiotics for animals?

- Do they use herbal medicine (standardized or not) as an alternative to antibiotics for animals? Who advised them? Where do they buy it?

- *Did the issues of antibiotic resistance come up in the previous discussion and if not* is he aware of this issue *(to be phrased in a way that the person understands without using this term)*, if so where did he learn about it, what does he think about it?

5) Discussion of the farm's pharmacy

- *Is there a pharmacy on the farm and if so,* is it possible to see it and discuss together about the products it contains? (Take one by one the different products and tell each other why it was bought, where, how much, why it can be used too, etc.)

If possible, take a picture of the pharmacy and where it is stored. List (on a sheet/book) the medicines in the pharmacy, note the molecules, the marketing names, the countries of manufacture, the producers, the wholesalers, describe the packaging; and extend the discussion from the interview.

Observe how the medicines are stored, what the pharmacy looks like, where it is stored, how the medicines are kept, what aspects they have (dirty, damaged...), etc.

Characteristics of the respondent *(to be filled in at the end of the interview, once the recorder is turned off, for information that will not be revealed during the interview)*

Level of education :

Age:

Renter/Lives in a relative's house/Owner:

Average monthly income: (from Observation, try to put the person in a category, 'rich', 'middle class', 'poor')

Mother tongue/sociolinguistic group/geographical origin (where was he/she born):

Religion, if Christian specify church:

Owns a vehicle? if yes, which one(s):

IMPROVING ANTIBIOTICS USE IN WEST AFRICA: EXPLORING CURRENT SITUATION AND DEVELOPING STRATEGIES FOR BEHAVIOUR CHANGE

Information sheet for household observations and interviews

Introduction

Dr. John Humphrey Amuasi is a principal investigator of this study. He is a senior research fellow and the Leader of the Global Health and Infectious Diseases Research Group at the Kumasi Centre for Collaborative Research. He is also a Lecturer at the Department of Global Health, School of Public Health at the Kwame Nkrumah University of Science and Technology. For further information about this study you may contact him via email on amuasi@kccr.de or by phone on +233 20 6300405.

I am a research assistant in sociology-anthropology and part of the collaboration of researchers from Germany, France, Burkina Faso and Ghana, conducting a study in this district on how to improve the health and wellbeing of members in the community

Background and study purpose

Our focus in this work is find out how people take care of their health and that of their children at home or by going to see health professionals, the way they use medicines, herbal products or anything else they use to take care of their health needs. I'm also interested in how people who raise animals take care of their health, by going to animal health professionals and giving them products. This work is part of a research project that is being conducted in Ghana and Burkina Faso. The objective is to propose improvements in health care in the countries and in particular in the use of antibiotics so that they remain effective against infections. The study will be conducted in accordance with the ethical principles defined in the Declaration of Helsinki as well as the Ghanaian regulatory requirements.

Study Procedure

[*The assembly man*].

In my discipline, we use a qualitative method which consists of staying in the community with the inhabitants, long enough to understand their life style and living conditions, by participating in the community life and freely discussing all sorts of subjects, but especially health issues, with the inhabitants. I would like to be able to stay for about several months and visit a number of communities within the district. I will make observations of distributors of health products, health centres, veterinarians or other animal health professionals and intensive or semi-intensive livestock farms near the communities, as well as of the community members, after having for permission. I will also conduct recorded in-depth interviews with various people living in the community and on the farms.

[*To the interviewee through in-depth interviews*].

The work will consist of a discussion with you that will last about 1h-1h30. To work better, I propose to record our conversation with a recorder. This is just to allow me to discuss without taking notes and to be in the discussion.

Risks and Benefits

All questions and observations will be appropriately phrased and conducted to avoid any risk of discomfort. You are free to accept or refuse to participate in this study and you may withdraw from the study at any time upon verbal notification. Your participation will however enable you share your knowledge and freely voice your opinion about issues and challenges you currently see around antibiotic use with the scientific community. This will help healthcare providers deliver improved and quality healthcare services.

Confidentiality

Our study guarantees the confidentiality of the participants' personal information. Anonymity will be respected, and we will conceal the identity of the individuals and the name of the community during the presentation of our work. Study data will be stored on our computer but your personal information will be anonymized from the start. We will use pseudonyms instead of people's names and we will not record your home address.

Voluntariness

Please be aware that your participation is completely voluntary. You may refuse to participate now, or at any time in time during the research. If you decide to discontinue your participation, it will not affect in any way the future treatment of you or other members of your community by health care workers

Withdrawal from the research

You may choose to withdraw from the research at any time without having to explain your decision to anyone. You may also choose not to answer any question you find it uncomfortable or private. Your withdrawal will have no consequences. You may also decide to withdraw during the administration of the interview. In that case, any information collected up to that point may still be used by the research team.

Cost/Compensation

If you agree to participate, can we make an appointment to do this interview. Can I take your phone number to confirm or if there is a problem? Thank you. The interview will be conducted over a snack. You are not required to make any contributions either in cash or in kind to this study. No monitory compensation will be granted for your participation. Your participation is therefore completely optional.

Outcome and feedback

The data collected will be analysed and presented as scientific reports for publication. Policy briefs will be developed and shared with stakeholders including Ghana Health Service.

Provision of Information and Consent for participants

If you agree to participate in this study a copy of the consent forms will be given to you to keep after you have signed or thumb printed. Permission to record the interview and take pictures

will be sought in the consent form. You have the right to agree or disagree. This will not affect you in anyway.

Sharing of participants Information/Data

Data generated from the study will be jointly owned by all members of the consortium and stored for six months after the study closure. Results of the study will however be published in scientific journals and also shared with other stakeholders including the Ghana Health Service.

Who to contact for further clarification/questions

If you have any questions, you can contact my supervisors at any time during the study:

Daniel Arhinful (darhinful@noguchi.ug.edu.gh – 024 4932139) and

Carine Baxerres (carine.baxerres@ird.fr – 0033 62769 0006).

If you have any concerns about the conduct of this study, your welfare or your rights as research participant, you may contact:

The Administrator

Ethical Review Committee

Research and Development Division

Ghana Health Service

P. O. Box MB 190

Accra

Tel: 0503539896

Email: ethics.research.ghsmail.org

IMPROVING ANTIBIOTICS USE IN WEST AFRICA: EXPLORING CURRENT SITUATION AND DEVELOPING STRATEGIES FOR BEHAVIOUR CHANGE

Information sheet for observations and interviews on intensive or semi-intensive farmers

Introduction

Dr. John Humphrey Amuasi is a principal investigator of this study. He is a senior research fellow and the Leader of the Global Health and Infectious Diseases Research Group at the Kumasi Centre for Collaborative Research. He is also a Lecturer at the Department of Global Health, School of Public Health at the Kwame Nkrumah University of Science and Technology. For further information about this study you may contact him via email on amuasi@kccr.de or by phone on +233 20 6300405.

I am a research assistant in sociology-anthropology and part of the collaboration of researchers from Germany, France, Burkina Faso and Ghana, conducting a study in this district on how to improve the health and wellbeing of members in the community

Background and study purpose

Our focus in this work is find out how people take care of their health and that of their children at home or by going to see health professionals, the way they use medicines, phytotherapy products or anything else they use to take care of their health. We're also interested in how people who raise animals take care of their health, by going to animal health professionals and giving them products. This work is part of a research project that is being conducted in Ghana and Burkina Faso. The objective is to propose improvements in health care in the countries and in particular in the use of antibiotics so that they remain effective against infections in both humans and animals. The study will be conducted in accordance with the ethical principles defined in the Declaration of Helsinki as well as the Ghanaian regulatory requirements.

Study Procedure

In my discipline, we use a qualitative method which consists in staying in the community, with the inhabitants long enough to understand their life style and living conditions, by participating in the life of the community and by freely discussing all kinds of subjects, but particularly health issues, with the inhabitants. We have obtained permission from the community chief/assembly man and a research permit from the Ghana Health Service. In addition to living and interacting with the inhabitants, we would like to meet and spend time with some of the professionals, like you, the livestock farmers. We have interacted with veterinarians, health distributors and other professionals at some of the community health centres in the district. I would therefore like to come and spend time with you and your work teams for 5 to 7 hours a day every day or every 2 days for about 2 months, either in the mornings or afternoons. I would like to see how your work goes, how you manage your farm, how the days are organized, how you take care of your animals, and what techniques and products you use. If you are willing, I will participate by carrying out some of your work activities and discuss it with your employees. I could do recorded interviews of 1 to 1.5 hours with you and some of them to clarify things I would not have understood.

Risks and Benefits

All observatory and participatory activities, and questions will be appropriately conducted and phrased to avoid any risk of discomfort to you, your workers, farm and farm animals, customers or partners. You are free to accept or refuse to participate in this study and you may withdraw from the study at any time upon verbal notification. Your participation will however enable you share your knowledge and freely voice your opinion about issues and challenges you currently see around antibiotic use with the scientific community. This will help healthcare providers deliver improved and quality healthcare services.

Confidentiality

Our study guarantees the confidentiality of the participants' personal information. Anonymity will be respected, and we will conceal the identity of the individuals and the name of the community during the presentation of our work. Study data will be stored on our computer but your personal information will be anonymized from the start. We will use pseudonyms instead of people's names and we will not record their home addresses.

Voluntariness

Please be aware that your participation is completely voluntary. You may refuse to participate now, or at any time in time during the research. If you decide to discontinue your participation, it will not affect in any way the future treatment of you or other members of your community by health care workers

Withdrawal from the research

You may choose to withdraw from the research at any time without having to explain your decision to anyone. You may also choose not to answer any question you find it uncomfortable or private. Your withdrawal will have no consequences. You may also decide to withdraw during the administration of the interview. In that case, any information collected up to that point may still be used by the research team.

Cost/Compensation

You are not required to make any contributions either in cash or in kind to this study. No monitory compensation will be granted for your participation. Your participation is therefore completely optional.

Outcome and feedback

The data collected will be analysed and presented as scientific reports for publication. Policy briefs will be developed and shared with stakeholders including Ghana Health Service.

Provision of Information and Consent for participants

If you agree to participate in this study a copy of the consent forms will be given to you to keep after you have signed or thumb printed. Permission to record the interview and take pictures will be sought in the consent form. You have the right to agree or disagree. This will not affect you in anyway.

Sharing of participants Information/Data

Data generated from the study will be jointly owned by all members of the consortium and stored for six months after the study closure. Results of the study will however be published in scientific journals and also shared with other stakeholders including the Ghana Health Service.

Who to contact for further clarification/questions

If you have any questions, you can contact my supervisors at any time during the study:

Daniel Arhinful (darhinful@noguchi.ug.edu.gh – 024 4932139) and

Carine Baxerres (carine.baxerres@ird.fr – 0033 62769 0006).

If you have any concerns about the conduct of this study, your welfare or your rights as research participant, you may contact:

The Administrator

Ethical Review Committee

Research and Development Division

Ghana Health Service

P. O. Box MB 190

Accra

Tel: 0503539896

Email: ethics.research.ghsmail.org

SOCIO-EPIDEMIOLOGY – INTERVIEW GUIDES

IDI Interview Guide for Community Members in Asante Akim North Municipality

**Respondent ID**	ID1 (Community Human Health)
Date of interview	
Age	
Gender	
Educational background	
Professional background	
Number of children	
Location (Agogo, Ananekrom)	

Narrative:

In-depth interview with community members in the Ashanti Region of Ghana on the understanding and perception of antibiotics and AMR.

This is a qualitative study designed to look into the understanding and perception of antibiotics and AMR among community members in the Ashanti Region of Ghana. The aim is to explore current knowledge on the topic of antibiotics and AMR, whether AMR is perceived as a problem and if so, what possible solutions against AMR could look like. The aim is to gain insights into perspectives of different community members.

Interviews will be conducted with patients/customers exiting pharmacies and chemical shops in Agogo and the health centre in Ananekrom. The number of interviews will be determined by the reaching of saturation. The results will form part of the ground research to develop an AMR intervention adapted to the local context and needs.

Objectives:

- To gain insights into community members' understanding and perception of antibiotics and AMR.
- To identify possible needs and challenges related to antibiotics and AMR.
- To identify possible solutions/interventions on AMR.

1. **Introduction**
   - Introduction of interviewer.
   - Explanation of project and interview procedure (participation voluntarily, not paid).
   - Signing of informed consent form.
2. **Introduction questions**
   - You have just excited the …. Did you get a prescription or antibiotics?
   - If not, do you still remember the last time you used antibiotics?
3. **. Antibiotics**
   - Do you know what antibiotics are and how would you describe them?

   [If the interviewee does not know what antibiotics are show them examples. Use toupaye/red-yellow pill]

   - According to you understanding, what is the purpose of antibiotics?

    - How would you describe the symptoms you have when you are taking antibiotics?
    - Can you know from the symptoms if you need antibiotics or not? Can the healthcare workers know? How do they know?
- Could you give me any examples of antibiotics you know or already used?
    - How do you identify whether a drug is an antibiotic? (name, packaging, colour)
    - Do you still remember what symptoms you experienced when you last used antibiotics?
- Do you still remember where you bought antibiotics when you last needed them?
    - Could you please describe the process of getting antibiotics?
    - Is it easy to get antibiotics? Where can you get them?
    - Could you describe any facilitators or barriers you might have experienced when last getting antibiotics?
    - Did it already happen to you that you wanted to have a specific medication, but the healthcare worker did not want to prescribe it to you?
- What is your experience with antibiotics? (positive/ negative, side effects, efficiency, instructions given)

4. AMR

- Have you heard about AMR already?

[If the interviewee does not know AMR explain it]

    - If yes, where and what?
    - How would you describe AMR?
- According to your opinion, what is causing and driving AMR?
    - Can you think of any practices that can lead to AMR? (human medicine, animal breeding, environment)
- How important do you think the topic of AMR is?
    - What are the dangers or problems related to AMR?
    - Does AMR have any consequences to you personally or your community? If so, which?
    - Does AMR have any consequences on animals or the environment? If so, which?
- According to your perception, is AMR discussed in Ghana? If yes, since when and how?
    - Do you think that AMR is national/regional/international problem? Why?

5. AMR action

- Have you heard or seen any campaign, pictures, radio messages, or other information about AMR? If so, what was the main message?
- What are do you think could be helpful to improve the situation regarding AMR?
    - Who should be involved in your suggested solutions?
    - Who should be targeted by such an intervention? (stakeholders, sectors)
- What role could you play in possible future action related to AMR?
- What role could your community play in possible future action related to AMR?

6. End/conclusion of interview

- Thank for time and valuable answers. Outlook.

IDI Interview Guide for Farmers in Asante Akim North Municipality

**Respondent ID**	ID1 (Community Animal Health)
Date of interview	
Age	
Gender	
Educational background	
Professional background	
Number animals (specify)	
Location	

Narrative:

In-depth interview with community members in the Ashanti Region of Ghana on the understanding and perception of antibiotics and AMR.

This is a qualitative study designed to look into the understanding and perception of antibiotics and AMR among community members in the Ashanti Region of Ghana. The aim is to explore current knowledge on the topic of antibiotics and AMR, whether AMR is perceived as a problem and if so, what possible solutions against AMR could look like. The aim is to gain insights into perspectives of different community members.

Interviews will be conducted with patients/customers exiting the wholesalers in Konongo and the informal veterinary market in Ananekrom. The number of interviews will be determined by the reaching of saturation. The results will form part of the ground research to develop an AMR intervention adapted to the local context and needs.

Objectives:

- To gain insights into farmers' understanding and perception of antibiotics and AMR.
- To identify possible needs and challenges related to antibiotics and AMR.
- To identify possible solutions/interventions on AMR.

1. **Introduction**
   - Introduction of interviewer.
   - Explanation of project and interview procedure (participation voluntarily, not paid).
   - Signing of informed consent form.
2. **Introduction questions**
   - You have just excited the …. Did you get antibiotics?
   - If not, do you still remember the last time you used antibiotics for your animals?
3. **. Antibiotics**
   - Do you know what antibiotics are and how would you describe them?

   [If the interviewee does not know what antibiotics are show them examples]

   - According to you understanding, what is the purpose of antibiotics?
     - Do you use them for preventive treatment or to cure sick animals?
     - How do you know when your animals need antibiotics?
     - Do you ask anyone for advice in such a case? If so, who?

    - How do they [veterinarians, veterinary technical assistants, others] know if your animals need antibiotics?
    - Can you know from the symptoms if your animals need antibiotics or not? Can the vets, vet technical assistants, or fellow farmers know? How do they know?
- Could you give me any examples of antibiotics you know or already used for your animals?
    - How do you identify whether a drug is an antibiotic? (name, packaging, colour)
- Do you still remember where you bought antibiotics when you last needed them for your animals?
    - Could you please describe the process of getting antibiotics?
    - Is it easy to get antibiotics? Where can you get them?
    - Could you describe any facilitators or barriers you might have experienced when last getting antibiotics?
- What is your experience with antibiotics? (positive/ negative, side effects, efficiency, instructions given, economic implications of adhering to withdrawal period)

4. AMR

- Have you heard about AMR already?

[If the interviewee does not know AMR explain it]

    - If yes, where and what?
    - How would you describe AMR?
- According to your opinion, what is causing and driving AMR?
    - Can you think of any practices that can lead to AMR? (human medicine, animal breeding, environment)
- How important do you think the topic of AMR is?
    - What are the dangers or problems related to AMR?
    - Does AMR have any consequences to your animals? If so, which?
    - Does AMR have any consequences for you personally or your community? If so, which?
- According to your perception, is AMR discussed in Ghana? If yes, since when and how?
    - Do you think that AMR is national/regional/international problem? Why?

5. AMR action

- Have you heard or seen any campaign, pictures, radio messages, or other information about AMR? If so, what was the main message?
- What are do you think could be helpful to improve the situation regarding AMR?
    - Who should be involved in your suggested solutions?
    - Who should be targeted by such an intervention? (stakeholders, sectors)
- What role could you play in possible future action related to AMR?
- What role could your community play in possible future action related to AMR?

6. End/conclusion of interview

Thank for time and valuable answers. Outlook.

Interview Guide Institutional Actors AMR – Animal Health

Interview Procedure

- Explain project and hand out flyer
- Introduce myself and explain my role in project → (1) understand viewpoints of actors with regards to AMR, (2) another project phase where actors will be involved in discussions about interventions
- Give consent form to read and sign and make clear whether they want to be identified (with name and/or institution) or want to be anonymized/de-identified

1) Before recording

- Profession, function, years of experience

2) AMR – General

- Perception of AMR & determinants of AMR, importance of AMR
- Consequences of AMR on work and animal sector
- AMR in Ghana: discussed since when, particular for Ghana/neighbouring countries
- Specific training received on AMR
- Description of institution's work and activities (involvement in prescription)
- Type of contact with farms

3) AMR – Animal Breeding

- Causes and drivers of AMR (human/vet. medicine, animal breeding, pharma industry)
- Knowledge and awareness of AMR in veterinary and farming sector
- Interconnection to human health (farm-to-fork-transmission) and environment (wastewater)
- Description of different farm management systems: IPC animal breeding, hygiene standards
- Guidelines/recommendations on AMU (legally binding?)
- Treatment of animals (who?)
- How, where, from whom to get antibiotics
- Prescription procedures & regulations
- Financing of antibiotics
- **Application** on AMU: How does it work? Current scope? Level of implementation?

4) AMR – Action

- Actors involved in AMR
- Interconnection between different levels (region, district, municipality)
- Actions taken on AMR: which and since when, effectiveness/obstacles, NAP
- Future action and needs
- Targeting of different stakeholder groups

Interview Guide Institutional Actors AMR – Environmental Health

Interview Procedure

- Explain project and hand out flyer
- Introduce myself and explain my role in project → (1) understand viewpoints of actors with regards to AMR, (2) another project phase where actors will be involved in discussions about interventions
- Give consent form to read and sign and make clear whether they want to be identified (with name and/or institution) or want to be anonymized/de-identified

1) Before recording

- Profession, function, years of experience

2) AMR – General

- Perception of AMR & determinants of AMR, importance of AMR
- Consequences of AMR on work and environmental sector (water)
- AMR in Ghana: discussed since when, particular for Ghana/neighbouring countries
- Specific training received on AMR

3) AMR – Environment/water

- Causes and driver of AMR (human/veterinary medicine, animal breeding, pharmaceutical industry)
- Knowledge and awareness of AMR in environmental health/water sector and general population
- Role of environment and interconnection to human health and veterinary sector (Transmission between human, animal sector? Impact on AMR?)
- Surveillance of sewage (antibiotics, AMR bacteria, high bacterial count, spread of water-borne diseases and AMR)
- Wastewater treatment plants
- Guidelines/recommendations for wastewater treatment plants

4) AMR – Action

- Actors involved in AMR, collaboration with other institutions
- Actions taken on AMR: which and since when, effectiveness/obstacles, NAP
- Future action and needs
- Targeting of different stakeholder groups

Interview Guide Institutional Actors AMR – Human Health

Interview Procedure

- Explain project and hand out flyer
- Introduce myself and explain my role in project → (1) understand viewpoints of actors with regards to AMR, (2) another project phase where actors will be involved in discussions about interventions

1) Before recording
- Profession, function, years of experience

2) AMR – General
- Perception and importance of AMR & determinants of AMR, importance of AMR
- Determinants and influences of AMR (e.g., WASH, SES)
- Causes and drivers of AMR (human/veterinary medicine, animal breeding, pharmaceutical industry)
- Consequences of AMR on work and health care sector
- AMR in Ghana: discussed since when, particular for Ghana/neighbouring countries
- Description of institution's work and activities
- Specific training received on AMR

3) AMR – Health care sector
- Knowledge and awareness of AMR among general population
- Knowledge and awareness of AMR in health care sector
  - Doctors, physician assistants, nurses, pharmacists, licensed drug sellers
  - AMR part of education/trainings?
- How, where, from whom to get antibiotics
  - Access vs. excess: where and how to get antibiotics → different levels (licensed over the counter, hospital pharmacy, etc.)
  - Prescription process and regulations
  - Quality of generic antibiotics? Counterfeit/substandard medicines
  - Payment of antibiotics (NHIS)
- Healthcare facilities:
  - IPC, antimicrobial stewardship program
  - Diagnostic tools
  - Surveillance of disease and AMU

4) AMR – Action
- Actors involved in AMR & actions taken on AMR: which and since when, effectiveness/obstacles, NAP
- Future action and needs, possible interventions → stakeholder groups to target

Participant Information Sheet

AMR B-CHANGE

Improving Antibiotics Use in West Africa: Exploring Current Situation and Developing Strategies for Behaviour Change

Introduction

Dr. John H. Amuasi is a principal investigator of this study. He is a senior researcher and the leader of the Global Health and Infectious Diseases Research Group at the Kumasi Centre for Collaborative Research. He is also a lecturer at the Department of Global Health, School of Public Health at the Kwame Nkrumah University of Science and Technology. For further information about this study, you can contact him via email (amuasi@kccr.de) or by phone (+233 20 6300405).

I am a doctoral student at Heidelberg Institute of Global Health and part of the collaboration of researchers from Burkina Faso, Ghana, Germany, and France. The project is funded by the French and German national research agencies, and research authorization has been obtained from the Ghana Health Service.

Study Background and Aims

The study is conducted in the Nouna District, Burkina Faso, and the Asante Akim North District, Ghana. We investigate the perception and utilization of antibiotics, including sources of the drugs and reasons for utilization as well as perceptions related to antimicrobial resistances (AMR) among health care workers, farmers, and communities. All data collected will serve to develop and refine interventions to improve awareness of antimicrobial resistance and use of antibiotics through effective communication, education, and training, and to stimulate responsible use of antimicrobial agents in humans and animals. Therefore, a cross-disciplinary stakeholder-driven and participatory approach will be taken to ensure that actual needs and expectations are met, and that eventually acceptable, equitable, effective, and sustainable health solutions will be developed.

So far baseline data on AMR pathogens along the food chain has been assessed and an exhaustive inventory of antibiotics in circulation at national and local level has been developed. Through interviews the perceptions of antibiotics and their effects by lay and professional actors in the field of human and veterinary health have been analysed. Additionally, communities' knowledge and perceptions related to AMR have been measured, and antibiotics utilization in primary and secondary health facilities has been quantified. The study is conducted in accordance with the ethical principles defined in the Declaration of Helsinki and the Ghanian regulatory requirements.

Participant Involvement

We would like to conduct a semi-structured interview with you for about 60 minutes to get your insights and perception about antimicrobial resistance and steps your institution is taking on

this health problem. The semi-structure nature of the interview will offer the possibility to explore aspects of antimicrobial resistance that are relevant to you or your institution. To have a more natural discussion we would like to audio record and subsequently transcribe the interview if you agree.

Voluntariness and Withdrawal from the Research
Please be aware that your participation is completely voluntary. You are not required to make any financial contributions to this study and no monetary compensation will be granted for your participation. You may withdraw from the study at any time without having to explain your decision. Moreover, you may choose to not answer questions. If you may decide to withdraw during the interview, any information collected up to that point may still be used by the research team.

Storage and Usage of Data
Our study guarantees the confidentiality of participants' personal information. Anonymity will be respected, and your identity will be concealed during the presentation of our work. Interview recordings will be deleted after transcription. To protect your privacy all data will be de-identified through de-identification of names and personal details in the transcription and the presentation of our work. Personal data relevant for the research (i.e., profession and affiliated institution) will be provided if written consent is given before the start of the interview. The transcripts will be stored for six months after the study closure on an external password coded hard drive that is only accessible by the research consortium. The collected data will be analysed and presented as scientific reports for publication. At the end of the research policy briefs will be developed and shared with stakeholders, including the Ghana Health Service.

Provision of Information and Consent
If you agree to participate in this study, a copy of the consent form will be given to you to keep after you have signed of thumb printed. Permission to record the interview and take pictures will sought in the consent form and you may choose to agree or disagree.

Further Information
If you have any further questions, you can kindly contact myself, Maresa Neuerer (maresa.neuerer@uni-heidelberg.de), or my supervisor Aurélia Souares (aurelia.souares@uni-heidelberg.de; +49 (0) 6221 56-5044) at any time.

In case of concerns regarding the conduct of this study, your welfare, or your rights as a research participant, you may contact:

The Administrator
Ethical Review Committee
Research and Development Division
Ghana Health Service
P.O. Box MB 190
Accra
Tel.: 0503539896
E-Mail: ethics.research@ghsmail.org
